# Supplementary material for: A behavioral signature for quantifying the social value of interpersonal relationships with specific others
Source: Commun Psychol. 2024 Sep 6;2:84. doi: 10.1038/s44271-024-00132-2 (PMC11379851; doi:10.1038/s44271-024-00132-2)
Supplement: Supplementary file 2 — Supplementary Information [file 44271_2024_132_MOESM2_ESM.pdf]

A Behavioral Signature for Quantifying the Social Value of Interpersonal Relationships with Specific Others - Guassi Moreira & Parkinson

Supplement

Methods

Extended MaxDiff Rationale. Intuitively, it might appear the most straightforward way to derive such a behavioral signature would simply be to collect Likert scale ratings on each of the activities. However, defining a behavioral signature according to our aims necessarily involves (i) deriving value weights for each activity by pitting the utility of activities against each other (thus incorporating opportunity cost) and (ii) estimating said weights such that they reflect the relative *scaling* between activities (i.e., the values of the weights describe the relative degree of preference between activity). These conditions implicitly rule out the use of Likert scale ratings because Likert scales are particularly susceptible to scale bias and are not easily able to accommodate trade-offs between activities that would be required to incorporate opportunity cost (34–36). Relatedly, asking participants to make forced choices between all possible activity pairings would be onerously time consuming and render the data susceptible to respondent fatigue.

For these reasons, we opted to use a MaxDiff design. Originally developed for use in marketing research, MaxDiff designs present respondents with a set of items and ask them to select the most appealing ('best') and least appealing ('worst') item based on a given feature of interest. This could involve a grocery chain polling potential customers about prospective seasonal snacks, or a polling company asking potential voters what issue they feel is most or least urgent for the current government to address. MaxDiff designs have enjoyed enormous popularity in marketing and applied economics (Sawtooth Software, 2020; Flynn, Terry, & Marley, 2012), and have also been creatively deployed in other disciplines (such as psychophysics, Maloney & Yang, 2003).

Critically, for our purposes, the competing nature of the MaxDiff design forces respondents to reveal trade-offs between items. Asking one to identify the 'best' and 'worst' items from a set theoretically requires individuals to evaluate every possible pair of items based on their subjective utilities and then select the most discrepant pairing. Combining this design with explicit instructions that emphasize scarcity should better align the ensuing activity weights with an economic concept of value (i.e., based on opportunity cost).

Number of Sets Used in the MaxDiff Study. As previously noted, we sourced two independent pools of activities from different populations to evaluate the robustness of our method. Following common guidelines (Sawtooth Software, 2020), the number of sets presented to participants was set to $n * (K/k)$, where n represents the number of times each activity is expected to appear across all sets, K represents the total number of items, and k represents the number of activities presented in each set. In our study, n was always set to 3, k was always set to 4, and K varied between the two different activity pools, 56 or 70, resulting in the presentation of 53 or 42 sets[1].

Extended Descriptions of Study Measures.

[1] A few additional 'screener' sets with attention checks were also presented to ensure participants were completing the experiment in a thoughtful manner.

Relationship Quality. The Inventory of Parent and Peer Attachment (IPPA; Armsden & Greenberg, 1987) was used to assess relationship quality in most of the exploratory data and all of the confirmatory data. We have extensively used the IPPA in prior similar studies to measure relationship quality (e.g., Guassi Moreira et al., 2018, 2021). Participants were instructed to use a 5-point Likert scale (1 = *almost never or never*, 5 = *almost always or always*) to answer 28 items about their relationship with their parent and a second set of 25 items about their relationship with their friend and, separately, their acquaintance (sample parent item: "My parent respects my feelings"; sample friend/acquaintance item: "When we discuss things, my friend considers my point of view"). Thus, participants completed the parent version once, and the friend version twice (the term 'friend' was replaced with 'acquaintance' in the items when asking about the latter). Responses were reverse-scored where appropriate and averaged to create a single composite score for relationship quality with each social partner. Reliability was good to excellent in both the exploratory (parent ω-total = 0.97, friend ω-total = 0.96, acquaintance ω-total = 0.82) and confirmatory (parent ω-total = 0.98, friend ω-total = 0.95, acquaintance ω-total = 0.95) samples.

As an added robustness check, we collected two additional measures of relationship quality in a subset of the exploratory data (N = 75) to ensure the association between social value scores and relationship quality could generalize to other instruments. The first was the Unidimensional Relationship Closeness Scale (URCS; Dibble et al., 2011), an 11-item measure that requires participants to answer questions about a given other on a 7-point Likert scale (1 = *Strongly disagree*, 7 = *Strongly agree*; sample items: "My [other] and I have a strong connection", "When we are apart, I miss my [other] a great deal"), and the second was the 7-item Relationship Assessment Scale (RAS; Hendrick, 1988) that involves a 5-point Likert scale (anchors vary; sample items: "How well does your [other] meet your needs?", "How many problems are there in your relationship?"). Both measures displayed good reliability (*RAS*: parent ω-total = 0.95, friend ω-total = 0.92, acquaintance ω-total = 0.94; *URCS*: parent ω-total = 0.98, friend ω-total = 0.97, acquaintance ω-total = 0.98). Items for both measures were reverse-scored where appropriate and averaged to yield relationship scores for each parent, friend, and acquaintance. Scores from both instruments correlated with social value scores in a similar manner as other relationship quality metrics, suggesting our procedure for quantifying social value yields scores that are generalizable with respect to various different instruments of relationship quality.

Big Five Personality Traits. We collected data on big five personality traits (Goldberg, 1993) of the activity generation (AG) and MaxDiff (MD) samples to characterize our samples. Personality traits were assessed by asking participants to complete the 44-item version of the Big Five Inventory (BFI-44; John & Srivastava, 1999). Participants were asked to indicate how well a series of items described themselves (7-point Likert scale, 1 = *Strongly disagree,* 7 = *Strongly agree*). Each item tapped one of five personality trait continua (Extraversion: "Is outgoing, sociable"; Agreeableness: "Is helpful and unselfish with others"; Conscientiousness: "Makes plans and follows through with them"; Neuroticism: "Gets nervous easily"; Openness: "Likes to reflect, play with ideas"). Items for each trait were reversed scored and averaged together to produce five scores per subject. We observed excellent reliability with the measure in our samples (AG ω-total = 0.95; MD ω-total = 0.95).

UCLA Loneliness Scale. Like the BFI, data on self-reported loneliness were also collected in the MD sample to better characterize samples. Loneliness was assessed using

Version 3 of the UCLA Loneliness Scale (Russell, 1996). The measure is comprised of twenty questions designed to assess subjective levels of loneliness, such as ‘How often do you feel there is no one you can turn to?’ and ‘How often do you feel left out?’. Participants used a four-point Likert scale to endorse each item (1 = “Never”, 4 = “Always). Reverse-worded items were recorded so that greater endorsement indicated greater loneliness. Scores were averaged after reverse coding to yield one value per participant. We observed excellent reliability with the measure in our sample (MD ω-total = 0.96).

One-Shot Dictator Game. Participants completed three separate one-trial dictator games (Fehr & Camerer, 2007) involving all possible pairings of the three social partners assessed in this study. Dictator games have been used in behavioral economics and social psychology to study fairness, altruism, and social preferences. Here we used the task as a vehicle for understanding social decision preferences involving specific social partners. Participants were presented with the following prompt. “*Suppose you had $100 to split between the [PERSON 1] and the [PERSON 2] you nominated. Use the dropdown box below to indicate how you would allocate the money”.* “PERSON 1” and “PERSON 2” were replaced with (parent, friend), (parent, acquaintance), and (friend, acquaintance) on their respective trials. Allocations ranged from ($100 PERSON 1, $0 PERSON 2) to ($0 PERSON 1, $100 PERSON 2) in $5 increments for a total of 21 choice options. Participants used a drop-down menu to select their preferred allocation. To aid interpretability during analysis, responses were recoded as the percentage of money allocated to PERSON 1 for a given trial (PERSON 1 = parent in [parent, friend] pair; PERSON 1 = parent in [parent, acquaintance] pair; PERSON 1 = friend in [friend, acquaintance] pair).

Forced Choice Question About Spending Time. Participants completed three separate forced choice questions about spending time with pairs of social partners. Participants were presented with the following prompt. “*Suppose you had a free afternoon with no obligations or commitments. Assuming you could only spend it with one person, would you rather spend it with [PERSON 1] you nominated or [PERSON 2] you nominated?”.* “PERSON 1” and “PERSON 2” were replaced with (parent, friend), (parent, acquaintance), and (friend, acquaintance) on their respective questions. The goal of this measure was to examine social decision-making preferences among these social partners when social outcomes (e.g., time spent with someone) were at stake (as opposed to monetary outcomes). Responses were binary coded among the pairs such that (1 = parent, 0 = 1 friend; 1 = parent, 0 = acquaintance; 1 = friend, 0 = acquaintance).

Social Loss Aversion. Participants completed a novel measure of what we termed ‘social loss aversion’. Participants used a slider ranging to 0 (not at all) to 100 (extremely) to indicate how upset they would be if they could no longer spend time with a given social partner. The question was presented to participants 3 times, once involving each social partner.

Ideal and Actual Time Spent. Participants were asked both (i) how often they saw and (ii) how often they wished to see each of the social partners on an average month. Participants used a dropdown menu to select the number of days, ranging from 0 to 30+ in increments of 1 day. The question was presented to participants 3 times, once involving each social partner.

Multi-Trial Social Decision Behavior. We used a pair of computerized delay discounting tasks to assess social decision preferences among the targets studied here. Our rationale for doing so is threefold. First, discounting decisions are both pervasive in everyday life and are

thought to be important for shaping life adjustment outcomes (Ludwig et al., 2019). Second, computerized discounting tasks deployed in research settings are flexible in their configuration, allowing researchers to study social decision behavior with respect to diverse reward outcomes (Seaman et al., 2016). Last, we previously used discounting tasks to reliably tap social decision preferences involving specific social partners (Guassi Moreira et al., 2021), thereby providing the current study with a baseline with which to provide a point of comparison.

Participants were asked to make decisions across two separate runs of a delay discounting task. On each trial, participants were presented with two hypothetical scenarios that pitted outcomes for two targets against each other. One scenario involved a relatively immediate, smaller reward and the other involved a relatively delayed, larger reward. The delays could take the value of zero (‘Today’), 2 weeks, 4 weeks, and 6 weeks. Values of zero and 6 weeks were never presented in the delayed or immediate scenarios, respectively. Both runs involved trials that pitted outcomes associated with each target against the other (parent vs friend outcomes; parent vs acquaintance outcomes; friend vs acquaintance outcomes). Because a given target could be associated with the immediate or delayed reward, this resulted in six unique conditions for the task (parent ~ immediate vs friend ~ delayed, friend ~ immediate vs parent delayed, etc.).

The two runs differed in the type of rewards offered to participants. One of the runs offered participants hypothetical monetary rewards (in USD) to be earned on behalf of either target (e.g., $16 for a given target); the other run was comprised of social rewards, offering participants hypothetical time spent with either target (e.g., 16 minutes of time spent with a given target). Values for each type of reward ranged from 2 – 30. There were 49 unique combinations of reward and time pairings (e.g., $2 now versus $18 two weeks from now) for each condition, resulting in 294 unique possible trial types (consistent with our prior work; Guassi Moreira et al., 2021). In the interest of reducing task demands for participants, each run was comprised of 60 trial types randomly selected from the master list of 294. Participants made their selection via button press on a keyboard and were granted as much time as needed to do so.

Both runs were programmed in PsychoPy3 (Peirce, 2007) and hosted on the online data collection platform Pavlovia.org. The visual characteristics of the task were programmed to be consistent with a prior study (Seaman et al., 2016). Participants accessed each run of the task through a link embedded in the Qualtrics survey. Importantly, participants received extensive instructions on how to complete the task and could not access the task link until confirming they understood the task. It was heavily stressed that participants were to complete the task as if all rewards were real, even though they were in fact hypothetical.

Modeling of Multi-Trial Social Decision Behavior. We used a hierarchical Bayesian model to analyze data from the multi-trial social decision tasks. Decisions on the i-th trial from the j-th participant on the discounting tasks were modeled as being distributed Bernoulli.

$$\text{Decision}_{ij} \sim \text{Ber}(p_{ij})$$

The distribution takes a single parameter (p) that describes the probability of ‘success’. Here, p_{ij} represents the probability of the j-th participant making a discounting decision (i.e., choosing the more immediate reward) on the i-th trial. The log odds of these probabilities were further modeled as a linear combination of trial-level variables.

$$\ln(p_{ij} / (1 - p_{ij})) = b_{1j}*PF_{ij} + b_{2j}*FP_{ij} + b_{3j}*PA_{ij} + b_{4j}*AP_{ij} + b_{5j}*FA_{ij} + b_{6j}*AF_{ij} + b_{7j}*RewardRatio_{ij}$$

In this parameterization, $b_{1j} – b_{6j}$ represent participant-specific decision preferences for choosing to, or to not, discount for a given condition on the task. Their corresponding variables are a set of overparameterized dummy codes that signify which condition corresponded to the i-th trial for the j-th participant. The first letter in each code designates which target was affected by the discounting decision (i.e., associated with the relatively immediate reward) and the second letter indicates the target affected by the non-discounting choice (associated with the relatively delayed reward). Thus, the PF_{ij} variable is a vector of 1s and 0s, with the former corresponding to the trials where the participant had to choose between a relatively immediate reward for a parent or a relatively delayed reward for a friend, and the latter corresponding to trials where this was not the case. The final coefficient, b_{7j}, corresponded to the effect of a ratio between the delayed reward over the immediate reward, and served as a control variable. Note that the lack of an intercept in this model is intentional, as it allows for estimation of adjusted means for each condition which denote the log odds of choosing to discount under each condition.

We entered our social value scores as moderators at the between-participantlevel such that scores for a given target moderated any trial-level effect involving that target:

$$b_{1j} = \gamma_{10} + \gamma_{11}*SocValPar_j + \gamma_{12}*SocValFri_j + u_{1j}$$

$$b_{2j} = \gamma_{20} + \gamma_{21}*SocValPar_j + \gamma_{22}*SocValFri_j + u_{2j}$$

$$b_{3j} = \gamma_{30} + \gamma_{31}*SocValPar_j + \gamma_{32}*SocValAcq_j + u_{3j}$$

$$b_{4j} = \gamma_{40} + \gamma_{41}*SocValPar_j + \gamma_{42}*SocValAcq_j + u_{4j}$$

$$b_{5j} = \gamma_{50} + \gamma_{51}*SocValFri_j + \gamma_{52}*SocValAcq_j + u_{5j}$$

$$b_{6j} = \gamma_{60} + \gamma_{61}*SocValFri_j + \gamma_{62}*SocValAcq_j + u_{6j}$$

Where 'SocVal' refers to a grand-mean centered social value score and 'Par' refers to parent, 'Fri' refers to friend, and 'Acq' refers to acquaintance. These terms were also entered into the model as lower-level predictors to ensure the validity of the higher order interaction term. The final trial-level coefficient (b_{7j}) was not associated with any moderating variables and thus would only be expressed as $b_{7j} = \gamma_{70} + u_{7j}$. Coefficients b_{1j} - b_{6j} represent the log odds of choosing to discount or not for a particular condition (e.g., when parent is affected by the discounting choice and friend is affected by the delayed choice) and thus coefficients for the cross-level interactions reflected changes in the log odds of choosing to discount or not in a particular condition. Each coefficient on its own cannot speak to the relationship between social value and a preference for a specific relationship partner across both conditions involving said partner. In order to fully understand the relationship between social decision preferences and social value we therefore needed to difference the posterior samples for coefficient pairs (γ_{11}, γ_{21}), (γ_{12}, γ_{22}), (γ_{31}, γ_{41}), (γ_{32}, γ_{42}), (γ_{51}, γ_{61}), and (γ_{52}, γ_{62}). The posterior means and credible intervals for these differences were used to understand the relationship between social value and social decision preferences. An additional version of the model without any between-participant predictors was run to replicate and extend prior work observed in Guassi Moreira et al., 2021 (results replicate prior work, not reported for sake of brevity). All hierarchical models were fit using the *brms* package in R (Bürkner, 2017) with a target average acceptance proposal probability of .95, a step-size of

.05, max tree depth of 15, 8 chains, 1000 iterations per chain with 500 warm-up samples and no thinning.

Affiliative Social Behavior Items. Participants were presented with additional forced choice items about affiliative social behavior in the confirmatory phase of the study. These items were included with the aim of enriching our understanding the association between social value scores and self-reported social behavior. The following items were presented to participants: 'Who are you more likely to turn to if you're seeking advice or support on an issue?', 'Who would you be more likely to celebrate something with?', 'Who is more likely to be the *first* person you would share positive personal news with?', 'Who is more likely to be the *first* person you would share negative personal news with?', 'Assuming both others needed to borrow money, but you could only lend to one person, who would you choose to lend money to?', 'Who would you rather go out to dinner with?'. Participants answered each question three times, covering all possible pairings of the social agents assessed in this study.

Pre-Registration Deviations. We initially pre-registered that we would control for relationship quality in all analyses. As the study progressed, following discussions with colleagues, we came to believe that this choice was misguided for a few reasons. First, it inadvertently implies a competition between relationship and quality and social value that we feel is inappropriate. How social value relates to the current study's outcome variables remains an open question, and whether or not relationship quality is a *better* predictor of these variables is not relevant to our purposes. Second, relationship quality is not privileged over other correlates of social value, and controlling for relationship quality would imply that we would need to control for all other correlates of social value when modeling the association between social value and social behavior, which is outside the scope of this manuscript, would inflate the manuscript's analytic flexibility, and might hamper the interpretability of our findings.

At the advice of peer reviewers, however, we ended up running *post hoc* analyses that included social values scores, relationship quality, and social loss aversion in the same model. The decision to do this was made after the initial deviation from the pre-registration to not control for relationship quality. All results from the *post hoc* analyses section were motivated by reviewer comments and were thus not part of our initial pre-registration. A final deviation was already noted in the manuscript regarding us beginning our confirmatory data collection but then switching from MTurk to Prolific due to a high incidence of fraudulent responding on MTurk. We analyzed and reported the usable MTurk data as tests of generalizability between online populations and replicability, more generally (and because it also seemed like good scientific practice to analyze usable data where available).

Results

Exploratory Phase

Paired Difference Analyses. Paired differences were estimated in the Cohen's *d* effect size metric and received a weakly informative prior (see code; osf.io/4qe7t). Overall, across the five exploratory subsamples, social value scores tended to be greater for friends relative to parents (Cohen's $d_{\text{Parent – Friend}}$: -0.13, moderate to robust evidence observed in 3/5 subsamples), parents relative to acquaintances (Cohen's $d_{\text{Parent – Acquaintance}}$: 0.54, robust evidence observed in 5/5 subsamples) and friends relative to acquaintances (Cohen's $d_{\text{Parent – Friend}}$: 0.64, robust evidence observed in 5/5 subsamples).

As a comparison, we also computed paired differences between relationship quality scores involving the three social partners. The rationale for this was to determine how the pattern of results with our metric of social value compares to another related yet distinct construct. Across the five exploratory subsamples, relationship quality was greater for friends relative to parents (Cohen's $d_{\text{Parent – Friend}}$: -0.62, robust evidence observed in 5/5 subsamples), parents relative to acquaintances (mean Cohen's $d_{\text{Parent – Acquaintance}}$: 0.36, moderate to robust evidence observed in 4/5 subsamples) and friends relative to acquaintances (mean Cohen's *d* $d_{\text{Parent – Friend}}$: 1.17, robust evidence observed in 5/5 subsamples). See Supplementary Table 7 for sample-specific results. An example of these general trends, using data from the confirmatory sample, is depicted in Supplementary Figure 2.

That the pattern of results involving social value scores resembled, but did not completely mimic, results with relationship quality suggests social value scores differentiate between social partners but may also provide unique information.

Confirmatory Phase

Paired Difference Analyses. As in the exploratory sample, social value scores tended to be greater for parents relative to acquaintances (mean Cohen's *d*: 0.51, robust evidence observed in 4/4 subsamples), and greater for friends relative to acquaintances (mean Cohen's *d*: 0.6, robust evidence observed in 4/4 subsamples). The evidence involving paired differences in social value scores for parents and friends was more mixed, with two subsamples robustly replicating the previously observed trend of friend > parent (mean Cohen's *d*: -0.31), and the other two subsamples suggesting the opposite effect (mean Cohen's *d*: 0.12), albeit with more modest evidence (smaller effect size, one confidence interval crosses zero).

Some relationship quality results here replicated findings from the exploratory phase. We again found that relationship quality was greater for friends relative to parents (mean Cohen's *d*: -0.76, robust evidence in 4/4 subsamples) and greater for friends relative to acquaintances (mean Cohen's *d*: 1.02, robust evidence in 4/4 subsamples). However, relationship quality was relatively more equivocal between parents and acquaintances, with a weaker preference observed for parents over acquaintances this time (mean Cohen's *d*: 0.07, moderate to robust evidence in 3/4 subsamples).

298 *Supplementary Table 1.* SONA-Generated Activities and SONA-Rated Weights

Activity	Raw Weight	Activity Variance	Scaled Weight
Self-applying a cosmetic treatment (ex: painting nails, styling hair)	-0.08	1.28	-0.06
Receiving a cosmetic treatment (ex: hair styling) from a professional	-0.29	1.31	-0.22
Attend to your personal finances (ex: budgeting, investment planning)	-0.48	0.32	-1.50
Attending a musical event	-0.05	0.44	-0.11
Attending a sporting event	-0.76	0.95	-0.80
Attending a performance (ex: theater)	-0.17	0.58	-0.29
Attending an academic event (ex: a public lecture)	-0.38	0.85	-0.45
Attending a social gathering	0.67	0.94	0.71
Attending a school or work-related activity	0.22	1.05	0.21
Completing personal chores/tasks	0.93	1.78	0.52
Discussing personal matters	-0.09	0.58	-0.16
Exploring city spaces	0.38	0.49	0.78
Getting ahead on work	0.52	0.9	0.58
Getting your car serviced (ex: oil change, tire change)	-0.85	1.22	-0.70
Giving someone a ride	-0.4	0.91	-0.44
Going for a walk	0.44	0.37	1.19
Going out for a drink	-0.49	1.08	-0.45
Going out to eat	0.74	0.6	1.23
Going to a cafe	0.35	0.73	0.48
Grocery shopping	0.26	0.39	0.67
Hosting a social gathering	-0.11	0.57	-0.19
Listening to music	0.75	0.94	0.80

Looking for new housing (ex: searching rentals or properties on zillow, touring a property)	-0.72	1.03	-0.70
Looking up directions to a place you may need to get to	0.1	0.75	0.13
Ordering take-out	0.08	0.92	0.09
Participating in online games/activities	0.57	1.47	0.39
Participating in recreational games (ex: bowling, chess, D&D, Risk)	0.24	1.08	0.22
Participating in a religious activity	-1.02	3.2	-0.32
Taking photographs as part of a hobby	-0.33	0.37	-0.89
Planning a gathering for work (ex: conference, job panel)	-0.72	0.25	-2.88
Planning a social gathering	-0.14	0.47	-0.30
Planning a vacation	-0.3	0.77	-0.39
Playing a musical instrument	-0.15	1.22	-0.12
Playing a sport	0.2	1.47	0.14
Playing a video game	0	2.06	0.00
Practicing a new skill (ex: musical instrument, writing compute code)	0.1	0.82	0.12
Preparing food (ex: cooking, baking)	0.41	0.58	0.71
Getting ready for a social gathering	0.13	1.26	0.10
Processing an emotional event	-0.16	0.65	-0.25
Reading	0.33	0.85	0.39
Sleeping/taking a nap	0.79	0.97	0.81
Researching a new product before purchasing it	0.21	0.54	0.39
Resting or relaxing	1.14	1.24	0.92
Seeking out advice	0.02	0.53	0.04

Seeking out something that makes you laugh	0.83	0.7	1.19
Sharing or creating online content (ex: Blogging, TikTok videos)	-0.34	1.25	-0.27
Shopping	0.46	0.34	1.35
Spending time at a mall	0.2	0.82	0.24
Spending time at a park	0.28	0.32	0.88
Spending time at a pool	-0.05	1.15	-0.04
Spending time at an amusement park	-0.32	1	-0.32
Spending time on activities related to a club	0.28	1.02	0.27
Spending time at amusement parks	-0.3	1.13	-0.27
Spending time on artistic activities	0.09	1.15	0.08
Spending time on social media (ex: TikTok, Twitter, Instagram, Facebook)	1.12	1.03	1.09
Playing with a pet(s)	0.06	1.38	0.04
Learning something new	0.22	0.4	0.55
Visiting a funeral home	-1.81	0.61	-2.97
Visiting a gun range for recreational shooting	-1.18	2.79	-0.42
Visiting a museum	-0.4	0.86	-0.47
Visiting the local library	-0.97	0.47	-2.06
Volunteering	-0.05	1.04	-0.05
Waiting in line for something anticipated (ex: concert tickets, merchandise, national park pass)	-0.69	0.97	-0.71
Watching a movie in a theater	-0.04	0.38	-0.11
Watching something on a streaming service	0.87	1.42	0.61
Watching something online (ex: YouTube)	1.24	1.09	1.14

Watching sports	-0.67	1.17	-0.57
Watching television	0.12	0.6	0.20
Going for a run	-0.34	1.43	-0.24
Lifting weights	-0.44	2.31	-0.19

Note. SONA refers to the UCLA undergraduate psychology subject pool. SONA participants generated activities, which experimenters pared down into slightly more abstract descriptions, as described in the main text, to facilitate consolidation of activities across respondents. A separate sample of SONA participants rated the activities in a MaxDiff design to yield the data that were used to calculate the weights that were used to compute social value scores.

Supplementary Table 2. Illustrative example of a MaxDiff design matrix

Choice	Set	*Overparameterized Indicator Variables*					
		A1	*A2*	*A3*	*A4*	*A5*	*A6*
1	1	1	0	0	0	0	0
0	1	0	0	1	0	0	0
0	1	0	0	0	1	0	0
1	1	-1	0	0	0	0	0
0	1	0	0	-1	0	0	0
0	1	0	0	0	-1	0	0
0	2	0	0	0	1	0	0
1	2	0	0	0	0	1	0
0	2	0	0	0	0	0	1
0	2	0	0	0	-1	0	0
0	2	0	0	0	0	-1	0
1	2	0	0	0	0	0	-1

Note. 'Choice' refers to whether the hypothetical activity was selected in either the 'best' or 'worst' column. A1 – A6 represent overparameterized codes indicating whether the activity was presented in the 'best' (1) or 'worst' column (-1), or neither (0). 'Set' refers to the random grouping of activities included on a given page of the survey.

Supplementary Table 3. Association between social value scores and social decision preferences on one-shot tasks (exploratory sample).

Task	Social Partner Pairs	Person 1	Person 2
Dictator Game (Sona-1)	Parent (1), Friend (2)	-0.059 [-0.37, 0.24]	-0.092 [-0.42, 0.23]
Dictator Game (Sona-1)	Parent (1), Acquaintance (2)	0.015 [-0.30, 0.32]	0.034 [-0.27, 0.37]
Dictator Game (Sona-1)	Friend (1), Acquaintance (2)	0.079 [-0.23, 0.40]	-0.029 [-0.36, 0.28]
Dictator Game (Sona-2)	Parent (1), Friend (2)	0.084 [-0.39, 0.53]	-0.400 [-0.88, 0.05]
Dictator Game (Sona-2)	Parent (1), Acquaintance (2)	-0.183 [-0.62, 0.24]	-0.004 [-0.41, 0.43]
Dictator Game (Sona-2)	Friend (1), Acquaintance (2)	0.072 [-0.33, 0.50]	-0.184 [-0.59, 0.23]
Dictator Game (Sona-3)	Parent (1), Friend (2)	0.110 [-0.11, 0.35]	-0.156 [-0.38, 0.09]
Dictator Game (Sona-3)	Parent (1), Acquaintance (2)	0.021 [-0.17, 0.21]	-0.302 [-0.49, -0.11]
Dictator Game (Sona-3)	Friend (1), Acquaintance (2)	-0.029 [-0.23, 0.18]	-0.258 [-0.46, -0.06]
Dictator Game (MTurk-1)	Parent (1), Friend (2)	0.139 [-0.07, 0.34]	-0.492 [-0.70, -0.30]
Dictator Game (MTurk-1)	Parent (1), Acquaintance (2)	0.167 [-0.04, 0.36]	-0.297 [-0.48, -0.10]
Dictator Game (MTurk-1)	Friend (1), Acquaintance (2)	-0.194 [-0.42, 0.02]	-0.022 [-0.24, 0.20]
Forced Choice Time (Sona-1)	Parent (1), Friend (2)	-0.057 [-0.58, 0.59]	-0.139 [-0.74, 0.44]
Forced Choice Time (Sona-1)	Parent (1), Acquaintance (2)	-0.223 [-1.15, 0.77]	-0.288 [-1.22, 0.66]
Forced Choice Time (Sona-1)	Friend (1), Acquaintance (2)	0.007 [-1.24, 1.28]	-0.017 [-1.24, 1.17]
Forced Choice Time (Sona-2)	Parent (1), Friend (2)	-0.239 [-1.11, 0.59]	-0.330 [-1.20, 0.54]
Forced Choice Time (Sona-2)	Parent (1), Acquaintance (2)	0.331 [-0.69, 1.22]	0.331 [-0.54, 1.35]
Forced Choice Time (Sona-2)	Friend (1), Acquaintance (2)	0.748 [-0.42, 1.97]	0.126 [-1.05, 1.32]

Forced Choice Time (Sona-3)	Parent (1), Friend (2)	-0.009 [-0.50, 0.46]	-0.671 [-1.19, -0.17]
Forced Choice Time (Sona-3)	Parent (1), Acquaintance (2)	-0.188 [-1.12, 0.72]	0.162 [-0.74, 1.08]
Forced Choice Time (Sona-3)	Friend (1), Acquaintance (2)	-0.016 [-1.26, 1.16]	0.012 [-1.28, 1.16]
Forced Choice Time (MTurk-1)	Parent (1), Friend (2)	0.131 [-0.31, 0.59]	-0.629 [-1.12, -0.15]
Forced Choice Time (MTurk-1)	Parent (1), Acquaintance (2)	0.224 [-0.28, 0.74]	-0.180 [-0.69, 0.32]
Forced Choice Time (MTurk-1)	Friend (1), Acquaintance (2)	-0.010 [-0.76, 0.73]	0.217 [-0.54, 0.95]

Note. Each row in the table represents a model wherein social value scores from two social partners were entered to predict a social decision preference on either dictator game or forced choice time spent items. 'Task' refers to paradigm used to capture social decision preferences. 'Social Partner Pairs' refers to which social partners' social value scores were included as predictors in the analysis. Outcomes were coded such that a positive slope for Person 1 means increases in Person 1's social value score were related to a preference for Person 1 over Person 2 for the given task; a negative slope for Person 2 means increases in Person 2's social value score were related to a preference for Person 2 over Person 1. Unbracketed numbers in the 'Person [1-2]' columns reflect posterior means of single-level regression coefficients; bracketed numbers are 89% HDI posterior credible intervals. Data for these analyses were collected as part of the exploratory phase of the study. Coefficients for the forced choice time spent task reflect log-odds. Subsamples are listed in parentheses; 'Sona' or 'MTurk' reflect where the sample completing the likelihood ratings was recruited from. Sona-2 was administered MTurk-sourced activities. All other samples were administered activities sourced from a different sample within the same population.

Supplementary Table 4. Association between social value scores and social decision preferences on one-shot tasks (confirmatory sample).

Task (Subsample)	Social Partner Pairs	Person 1	Person 2
Dictator Game (Sona_MTurk)	Parent (1), Friend (2)	0.579 [0.36, 0.84]	-0.508 [-0.74, -0.26]
Dictator Game (Sona_MTurk)	Parent (1), Acquaintance (2)	0.284 [0.09, 0.47]	-0.205 [-0.40, -0.01]
Dictator Game (Sona_MTurk)	Friend (1), Acquaintance (2)	0.198 [0.00, 0.40]	-0.155 [-0.35, 0.04]
Dictator Game (MTurk_MTurk)	Parent (1), Friend (2)	0.172 [-0.00, 0.37]	-0.403 [-0.59, -0.22]
Dictator Game (MTurk_MTurk)	Parent (1), Acquaintance (2)	0.273 [0.11, 0.44]	-0.317 [-0.48, -0.15]
Dictator Game (MTurk_MTurk)	Friend (1), Acquaintance (2)	0.202 [0.02, 0.39]	-0.215 [-0.39, -0.02]
Dictator Game (Sona_Prolific)	Parent (1), Friend (2)	0.240 [0.12, 0.37]	-0.221 [-0.36, -0.10]

Dictator Game (Sona_Prolific)	Parent (1), Acquaintance (2)	0.205 [0.09, 0.31]	-0.262 [-0.37, -0.16]
Dictator Game (Sona_Prolific)	Friend (1), Acquaintance (2)	0.230 [0.13, 0.35]	-0.356 [-0.46, -0.25]
Dictator Game (MTurk_Prolific)	Parent (1), Friend (2)	0.201 [0.07, 0.32]	-0.070 [-0.19, 0.05]
Dictator Game (MTurk_Prolific)	Parent (1), Acquaintance (2)	0.248 [0.14, 0.36]	-0.103 [-0.22, 0.00]
Dictator Game (MTurk_Prolific)	Friend (1), Acquaintance (2)	0.167 [0.06, 0.29]	-0.233 [-0.34, -0.12]
Forced Choice Time (Sona_MTurk)	Parent (1), Friend (2)	0.570 [0.01, 1.10]	-1.132 [-1.74, -0.54]
Forced Choice Time (Sona_MTurk)	Parent (1), Acquaintance (2)	0.828 [0.28, 1.39]	-0.567 [-1.09, -0.06]
Forced Choice Time (Sona_MTurk)	Friend (1), Acquaintance (2)	-0.247 [-1.37, 0.89]	-0.794 [-1.73, 0.24]
Forced Choice Time (MTurk_MTurk)	Parent (1), Friend (2)	0.282 [-0.15, 0.64]	-0.743 [-1.17, -0.33]
Forced Choice Time (MTurk_MTurk)	Parent (1), Acquaintance (2)	0.962 [0.42, 1.42]	-0.654 [-1.11, -0.22]
Forced Choice Time (MTurk_MTurk)	Friend (1), Acquaintance (2)	0.393 [-0.21, 1.08]	0.117 [-0.57, 0.78]
Forced Choice Time (Sona_Prolific)	Parent (1), Friend (2)	0.449 [0.17, 0.71]	-0.480 [-0.75, -0.21]
Forced Choice Time (Sona_Prolific)	Parent (1), Acquaintance (2)	0.627 [0.29, 0.95]	-0.817 [-1.12, -0.46]
Forced Choice Time (Sona_Prolific)	Friend (1), Acquaintance (2)	0.269 [-0.30, 0.82]	-0.437 [-1.00, 0.10]
Forced Choice Time (MTurk_Prolific)	Parent (1), Friend (2)	0.171 [-0.09, 0.42]	-0.254 [-0.50, -0.00]
Forced Choice Time (MTurk_Prolific)	Parent (1), Acquaintance (2)	0.278 [-0.00, 0.59]	-0.359 [-0.65, -0.06]
Forced Choice Time (MTurk_Prolific)	Friend (1), Acquaintance (2)	0.224 [-0.29, 0.76]	-0.484 [-0.96, 0.01]

Note. Each row in the table represents a model wherein social value scores from two social partners were entered to predict a social decision preference on either dictator game or forced choice time spent items. ‘Task’ refers to paradigm used to capture social decision preferences. ‘Social Partner Pairs’ refers to which social partners’ social value scores were included as predictors in the analysis. Outcomes were coded such that a positive slope for Person 1 means increases in Person 1’s social value score were related to a preference for Person 1 over Person 2 for the given task; a negative slope for Person 2 means increases in Person 2’s social value score were related to a preference for Person 2 over Person 1. Unbracketed numbers in the ‘Person [1-2]’ columns reflect posterior means of single-level regression coefficients; bracketed numbers are 89% HDI posterior credible intervals. Data for these analyses were collected as part of the confirmatory phase of the study. Coefficients for the forced choice time spent task reflect log-odds. Subsamples are listed in parentheses; the first term denotes the population from which the activities were sourced; the second term denotes which population completed the likelihood ratings and other measures.

Supplementary Table 5. Social value scores and social decision preferences on the multi-trial social decision-making task (confirmatory sample)

Term	Monetary – SONA – Prolific	Social – SONA – Prolific	Monetary – MTurk – Prolific	Social – MTurk – Prolific
Parent vs Friend Preference x Parent Social Value	0.11 [0.07, 0.15]	0.08 [0.04, 0.11]	0.09 [0.06, 0.13]	0.07 [0.04, 0.10]
Parent vs Friend Preference x Friend Social Value	-0.09 [-0.12, -0.06]	-0.10 [-0.13, -0.06]	-0.05 [-0.08, -0.02]	-0.08 [-0.11, -0.05]
Parent vs Acquaintance Preference x Parent Social Value	0.15 [0.11, 0.19]	0.11 [0.08, 0.15]	0.09 [0.07, 0.13]	0.08 [0.05, 0.11]
Parent vs Acquaintance Preference x Acquaintance Social Value	-0.10 [-0.14, -0.06]	-0.11 [-0.14, -0.07]	0.00 [-0.03, 0.04]	-0.05 [-0.09, -0.01]
Friend vs Acquaintance Preference x Parent Social Value	0.10 [0.07, 0.12]	0.13 [0.10, 0.16]	0.06 [0.03, 0.09]	0.08 [0.05, 0.12]
Friend vs Acquaintance Preference x Acquaintance Social Value	-0.09 [-0.13, -0.06]	-0.12 [-0.16, -0.09]	-0.02 [-0.06, 0.01]	-0.05 [-0.09, -0.01]
Number of participants, Number of decisions	*N* = 214, *n* = 12,559	*N* = 210, *n* = 12,462	*N* = 211, *n* = 12,431	*N* = 200, *n* = 11,769

Note. Bracketed numbers are 89% HDI posterior credible intervals. Each entry into the table is a coefficient from the hierarchical model described in this supplement. Specifically, the coefficient is the cross-level interaction between a dummy code for trial type (indicating which relationship partner is affected by a discounting option and which partner is affected by a delayed option) and the social value score for a specific partner. Substantively, the coefficient represents the expected difference in the log-odds of a favoring a given relationship partner over another following a one-unit difference in the social value score. A positive slope was expected for any terms involving parent social value and thus indicates a parent-over-other preference strengthens with increasing social value for parent; a negative slope was expected for any terms involving acquaintance social value and thus indicates an acquaintance-over-other preference strengths with increasing social value for acquaintance; a negative slope was expected for friend social value when pitted against parent, and a positive slope was expected pitted against friend. The column headers describe first the type of reward at stake, followed by the sample from which the activities were sourced, followed by the sample that completed the likelihood ratings and other measures.

Supplementary Table 6. Social value scores and social decision preferences on the multi-trial social decision-making task (confirmatory sample)

Term	Monetary – (SONA – MTurk)	Social – (SONA – MTurk)	Monetary – (MTurk – MTurk)	Social – (MTurk – MTurk)
Parent vs Friend Preference x Parent Social Value	0.13 [0.06, 0.20]	0.19 [0.11, 0.26]	0.09 [0.04, 0.13]	0.10 [0.05, 0.16]
Parent vs Friend Preference x Friend Social Value	-0.10 [-0.16, -0.04]	-0.19 [-0.25, -0.12]	-0.09 [-0.13, -0.04]	-0.13 [-0.18, -0.07]
Parent vs Acquaintance Preference x Parent Social Value	0.13 [0.07, 0.20]	0.27 [0.16, 0.37]	0.16 [0.10, 0.22]	0.14 [0.08, 0.20]
Parent vs Acquaintance Preference x Acquaintance Social Value	-0.03 [-0.10, 0.04]	-0.18 [-0.30, -0.05]	-0.14 [-0.21, -0.07]	-0.08 [-0.16, -0.01]
Friend vs Acquaintance Preference x Parent Social Value	0.11 [0.05, 0.17]	0.19 [0.12, 0.25]	0.13 [0.07, 0.18]	0.14 [0.08, 0.20]
Friend vs Acquaintance Preference x Acquaintance Social Value	0.02 [-0.05, 0.10]	-0.08 [-0.16, 0.00]	-0.09 [-0.16, -0.02]	-0.09 [-0.16, -0.02]
Number of participants, Number of decisions	N = 67, n = 3,924	N = 67, n = 3,929	N = 92, n = 5,460	N = 85, n = 5,006

Note. Bracketed numbers are 89% HDI posterior credible intervals. Each entry into the table is a coefficient from the hierarchical model described in this supplement. Specifically, the coefficient is the cross-level interaction between a dummy code for trial type (indicating which relationship partner is affected by a discounting option and which partner is affected by a delayed option) and the social value score for a specific partner. Substantively, the coefficient represents the expected difference in the log-odds of a favoring a given relationship partner over another following a one-unit difference in the social value score. A positive slope was expected for any terms involving parent social value and thus indicates a parent-over-other preference strengthens with increasing social value for parent; a negative slope was expected for any terms involving acquaintance social value and thus indicates an acquaintance-over-other preference strengths with increasing social value for acquaintance; a negative slope was expected for

friend social value when pitted against parent, and a positive slope was expected pitted against friend. The column headers describe first the type of reward at stake, followed by the sample from which the activities were sourced, followed by the sample that completed the likelihood ratings and other measures.

Supplementary Table 7. Social value scores predict affiliative social behaviors (SONA-derived activities, MTurk sample).

Behavior	Social Partner Pairs	Person 1	Person 2
Advice	Parent (1), Friend (2)	0.713 [0.20, 1.27]	-1.462 [-2.05, -0.88]
Advice	Parent (1), Acquaintance (2)	0.998 [0.40, 1.54]	-0.963 [-1.46, -0.44]
Advice	Friend (1), Acquaintance (2)	0.846 [0.10, 1.68]	-0.556 [-1.29, 0.15]
Celebration	Parent (1), Friend (2)	0.184 [-0.33, 0.72]	-0.569 [-1.09, -0.03]
Celebration	Parent (1), Acquaintance (2)	0.953 [0.44, 1.51]	-0.508 [-0.94, -0.04]
Celebration	Friend (1), Acquaintance (2)	0.917 [0.06, 1.68]	0.474 [-0.30, 1.28]
Positive News	Parent (1), Friend (2)	1.144 [0.57, 1.73]	-1.230 [-1.77, -0.61]
Positive News	Parent (1), Acquaintance (2)	1.536 [0.85, 2.17]	-0.438 [-0.97, 0.08]
Positive News	Friend (1), Acquaintance (2)	0.700 [-0.09, 1.48]	0.282 [-0.46, 1.11]
Negative News	Parent (1), Friend (2)	0.687 [0.16, 1.17]	-0.643 [-1.22, -0.18]
Negative News	Parent (1), Acquaintance (2)	1.285 [0.66, 1.82]	-0.953 [-1.47, -0.48]
Negative News	Friend (1), Acquaintance (2)	1.180 [0.48, 1.98]	0.242 [-0.50, 1.00]
Lend Money	Parent (1), Friend (2)	1.108 [0.51, 1.67]	-0.590 [-1.12, -0.03]
Lend Money	Parent (1), Acquaintance (2)	1.786 [1.08, 2.51]	-0.632 [-1.17, -0.08]
Lend Money	Friend (1), Acquaintance (2)	0.761 [0.11, 1.53]	0.300 [-0.44, 1.05]
Have Dinner	Parent (1), Friend (2)	0.052 [-0.46, 0.66]	-0.634 [-1.18, -0.07]
Have Dinner	Parent (1), Acquaintance (2)	0.433 [-0.01, 0.90]	-0.795 [-1.29, -0.40]
Have Dinner	Friend (1), Acquaintance (2)	1.127 [0.41, 1.87]	-0.278 [-0.97, 0.36]

Note. Each row in the table represents a model wherein social value scores from two social partners were entered to predict a forced choice between the two social partners for a given type social affiliative behavior (e.g., which of the social partners would participants choose to seek advice from, be more likely to lend more to). ‘Behavior’ refers to the forced choice affiliative

behavior being predicted. The full text for each question can be accessed in the main document. ‘Person Pairs’ refers to which social partners’ social value scores were included as predictors in the analysis. Outcomes were binary and coded such that a positive slope for Person 1 means increases in Person 1’s social value score were related to choosing Person 1 over Person 2 for the given affiliative behavior; a negative slope for Person 2 means increases in Person 2’s social value score were related to choosing Person 2 over Person 1. Unbracketed numbers in the ‘Person [1-2]’ columns reflect posterior means of single-level regression coefficients; bracketed numbers are 89% HDI posterior credible intervals. Data for these analyses were collected as part of the confirmatory phase of the study. Coefficients reflect log-odds.

Supplementary Table 8. Social value scores predict affiliative social behaviors (MTurk-derived activities, MTurk sample).

Behavior	Social Partner Pairs	Person 1	Person 2
Advice	Parent (1), Friend (2)	0.241 [-0.15, 0.62]	-0.972 [-1.40, -0.54]
Advice	Parent (1), Acquaintance (2)	0.590 [0.13, 1.04]	-0.934 [-1.36, -0.49]
Advice	Friend (1), Acquaintance (2)	0.276 [-0.27, 0.92]	0.210 [-0.45, 0.84]
Celebration	Parent (1), Friend (2)	-0.007 [-0.37, 0.41]	-0.112 [-0.52, 0.25]
Celebration	Parent (1), Acquaintance (2)	1.204 [0.68, 1.68]	-0.712 [-1.15, -0.27]
Celebration	Friend (1), Acquaintance (2)	0.597 [-0.18, 1.31]	-0.175 [-0.88, 0.61]
Positive News	Parent (1), Friend (2)	0.343 [-0.06, 0.72]	-0.508 [-0.92, -0.13]
Positive News	Parent (1), Acquaintance (2)	0.824 [0.30, 1.29]	-0.561 [-1.02, -0.16]
Positive News	Friend (1), Acquaintance (2)	0.456 [-0.22, 1.03]	-0.294 [-0.92, 0.33]
Negative News	Parent (1), Friend (2)	0.287 [-0.13, 0.63]	-0.587 [-0.97, -0.19]
Negative News	Parent (1), Acquaintance (2)	0.583 [0.16, 0.95]	-0.786 [-1.21, -0.40]
Negative News	Friend (1), Acquaintance (2)	0.644 [0.04, 1.21]	-0.486 [-1.03, 0.05]
Lend Money	Parent (1), Friend (2)	0.741 [0.29, 1.17]	-0.735 [-1.17, -0.29]
Lend Money	Parent (1), Acquaintance (2)	0.932 [0.41, 1.43]	-0.730 [-1.18, -0.28]
Lend Money	Friend (1), Acquaintance (2)	0.317 [-0.25, 0.86]	-0.594 [-1.12, -0.04]
Have Dinner	Parent (1), Friend (2)	0.094 [-0.31, 0.53]	-0.638 [-1.07, -0.23]
Have Dinner	Parent (1), Acquaintance (2)	0.739 [0.31, 1.16]	-0.773 [-1.19, -0.37]
Have Dinner	Friend (1), Acquaintance (2)	0.895 [0.16, 1.65]	-0.504 [-1.21, 0.20]

Note. Each row in the table represents a model wherein social value scores from two social partners were entered to predict a forced choice between the two social partners for a given type social affiliative behavior (e.g., which of the social partners would participants choose to seek advice from, be more likely to lend more to). ‘Behavior’ refers to the forced choice affiliative behavior being predicted. The full text for each question can be accessed in the main document. ‘Social Partner Pairs’ refers to which social partners’ social value scores were included as predictors in the analysis. Outcomes were binary and coded such that a positive slope for Person 1 means increases in Person 1’s social value score were related to choosing Person 1 over Person 2 for the given affiliative behavior; a negative slope for Person 2 means increases in Person 2’s social value score were related to choosing Person 2 over Person 1. Unbracketed numbers in the ‘Person [1-2]’ columns reflect posterior means of single-level regression coefficients; bracketed numbers are 89% HDI posterior credible intervals. Data for these analyses were collected as part of the confirmatory phase of the study. Coefficients reflect log-odds.

Supplementary Table 9. Social value scores predict affiliative social behaviors (SONA-derived activities, Prolific sample).

Behavior	Social Partner Pairs	Person 1	Person 2
Advice	Parent (1), Friend (2)	0.323 [0.05, 0.57]	-0.147 [-0.41, 0.11]
Advice	Parent (1), Acquaintance (2)	0.570 [0.25, 0.91]	-0.758 [-1.07, -0.42]
Advice	Friend (1), Acquaintance (2)	-0.004 [-0.53, 0.53]	-0.479 [-0.93, 0.01]
Celebration	Parent (1), Friend (2)	0.510 [0.22, 0.79]	-0.304 [-0.59, -0.04]
Celebration	Parent (1), Acquaintance (2)	0.635 [0.28, 1.03]	-0.642 [-0.99, -0.27]
Celebration	Friend (1), Acquaintance (2)	0.212 [-0.51, 0.95]	-0.134 [-0.85, 0.61]
Positive News	Parent (1), Friend (2)	0.276 [0.03, 0.54]	-0.144 [-0.40, 0.12]
Positive News	Parent (1), Acquaintance (2)	1.119 [0.68, 1.52]	-0.587 [-1.01, -0.20]
Positive News	Friend (1), Acquaintance (2)	0.271 [-0.31, 0.88]	-0.024 [-0.64, 0.56]
Negative News	Parent (1), Friend (2)	-0.057 [-0.31, 0.19]	-0.127 [-0.38, 0.12]
Negative News	Parent (1), Acquaintance (2)	0.472 [0.17, 0.75]	-0.577 [-0.86, -0.28]
Negative News	Friend (1), Acquaintance (2)	0.658 [0.18, 1.13]	-0.541 [-1.02, -0.07]
Lend Money	Parent (1), Friend (2)	0.586 [0.28, 0.92]	-0.372 [-0.69, -0.06]
Lend Money	Parent (1), Acquaintance (2)	0.781 [0.36, 1.21]	-0.822 [-1.22, -0.39]
Lend Money	Friend (1), Acquaintance (2)	-0.218 [-0.67, 0.31]	-0.359 [-0.80, 0.10]
Have Dinner	Parent (1), Friend (2)	0.190 [-0.11,0.47]	-0.428 [-0.72, -0.15]
Have Dinner	Parent (1), Acquaintance (2)	0.486 [0.18, 0.78]	-0.990 [-1.28, -0.69]
Have Dinner	Friend (1), Acquaintance (2)	0.378 [-0.15, 0.88]	-0.728 [-1.21, -0.24]

Note. Each row in the table represents a model wherein social value scores from two social partners were entered to predict a forced choice between the two social partners for a given

type social affiliative behavior (e.g., which of the social partners would participants choose to seek advice from, be more likely to lend more to). 'Behavior' refers to the forced choice affiliative behavior being predicted. The full text for each question can be accessed in the main document. 'Social Partner Pairs' refers to which social partners' social value scores were included as predictors in the analysis. Outcomes were binary and coded such that a positive slope for Person 1 means increases in Person 1's social value score were related to choosing Person 1 over Person 2 for the given affiliative behavior; a negative slope for Person 2 means increases in Person 2's social value score were related to choosing Person 2 over Person 1. Unbracketed numbers in the 'Person [1-2]' columns reflect posterior means of single-level regression coefficients; bracketed numbers are 89% HDI posterior credible intervals. Data for these analyses were collected as part of the confirmatory phase of the study. Coefficients reflect log-odds.

Supplementary Table 10. Social value scores predict affiliative social behaviors (MTurk-derived activities, Prolific sample).

Behavior	Social Partner Pairs	Person 1	Person 2
Advice	Parent (1), Friend (2)	0.130 [-0.11, 0.37]	-0.248 [-0.48, 0.02]
Advice	Parent (1), Acquaintance (2)	0.113 [-0.19, 0.44]	-0.154 [-0.46, 0.16]
Advice	Friend (1), Acquaintance (2)	0.331 [-0.21, 0.86]	-0.221 [-0.75, 0.31]
Celebration	Parent (1), Friend (2)	0.122 [-0.12, 0.38]	-0.036 [-0.29, 0.22]
Celebration	Parent (1), Acquaintance (2)	-0.039 [-0.38, 0.32]	-0.231 [-0.56, 0.07]
Celebration	Friend (1), Acquaintance (2)	0.007 [-0.70, 0.74]	-0.378 [-1.03, 0.28]
Positive News	Parent (1), Friend (2)	0.452 [0.17, 0.72]	-0.345 [-0.61, -0.09]
Positive News	Parent (1), Acquaintance (2)	0.361 [-0.11, 0.84]	-0.252 [-0.72, 0.20]
Positive News	Friend (1), Acquaintance (2)	0.123 [-0.43, 0.61]	-0.360 [-0.83, 0.09]
Negative News	Parent (1), Friend (2)	0.245 [-0.02, 0.49]	-0.163 [-0.41, 0.08]
Negative News	Parent (1), Acquaintance (2)	0.058 [-0.23, 0.34]	-0.260 [-0.52, 0.02]

Negative News	Friend (1), Acquaintance (2)	0.130 [-0.29, 0.55]	-0.267 [-0.67, 0.10]
Lend Money	Parent (1), Friend (2)	0.637 [0.34, 0.94]	-0.373 [-0.65, -0.09]
Lend Money	Parent (1), Acquaintance (2)	0.137 [-0.30, 0.59]	-0.027 [-0.48, 0.45]
Lend Money	Friend (1), Acquaintance (2)	0.329 [-0.13, 0.81]	-0.461 [0.88, -0.01]
Have Dinner	Parent (1), Friend (2)	0.111 [-0.14, 0.34]	-0.126 [-0.37, 0.12]
Have Dinner	Parent (1), Acquaintance (2)	0.008 [-0.30, 0.31]	-0.200 [-0.47, 0.11]
Have Dinner	Friend (1), Acquaintance (2)	0.169 [-0.30, 0.62]	-0.451 [-0.87, -0.03]

Note. Each row in the table represents a model wherein social value scores from two social partners were entered to predict a forced choice between the two social partners for a given type social affiliative behavior (e.g., which of the social partners would participants choose to seek advice from, be more likely to lend more to). 'Behavior' refers to the forced choice affiliative behavior being predicted. The full text for each question can be accessed in the main document. 'Person Pairs' refers to which social partners' social value scores were included as predictors in the analysis. Outcomes were binary and coded such that a positive slope for Person 1 means increases in Person 1's social value score were related to choosing Person 1 over Person 2 for the given affiliative behavior; a negative slope for Person 2 means increases in Other 2's social value score were related to choosing Person 2 over Person 1. Unbracketed numbers in the 'Person [1-2]' columns reflect posterior means of single-level regression coefficients; bracketed numbers are 89% HDI posterior credible intervals. Data for these analyses were collected as part of the confirmatory phase of the study. Coefficients reflect log-odds.

Supplementary Table 11. Paired differences in social value scores and relationship quality scores (confirmatory sample)

Comparison (Subsample)	Social Value	Relationship Quality
Parent – Friend (Sona_MTurk)	-0.32 [-0.48, -0.16]	-0.70 [-0.88, -0.53]
Parent – Acquaintance (Sona_MTurk)	0.31 [0.15, 0.46]	0.01 [-0.15, 0.15]
Friend – Acquaintance (Sona_MTurk)	0.50 [0.32, 0.66]	0.88 [0.68, 1.05]

Parent – Friend (MTurk_MTurk)	0.12 [-0.03, 0.27]	-0.64 [-0.80, -0.48]
Parent – Acquaintance (MTurk_MTurk)	0.42 [0.27, 0.57]	0.07 [-0.09, 0.20]
Friend – Acquaintance (MTurk_MTurk)	0.38 [0.23, 0.53]	0.82 [0.67, 1.00]
Parent – Friend (Sona_Prolific)	-0.25 [-0.35, -0.15]	-0.79 [-0.90, -0.67]
Parent – Acquaintance (Sona_Prolific)	0.45 [0.35, 0.56]	0.02 [-0.08, 0.13]
Friend – Acquaintance (Sona_Prolific)	0.70 [0.58, 0.81]	0.98 [0.85, 1.10]
Parent – Friend (MTurk_Prolific)	0.17 [0.07, 0.28]	-0.77 [-0.89, -0.65]
Parent – Acquaintance (MTurk_Prolific)	0.75 [0.63, 0.88]	0.16 [0.05, 0.26]
Friend – Acquaintance (MTurk_Prolific)	0.60 [0.49, 0.72]	1.12 [0.98, 1.26]

Note. Brackets represent 89% HDIs of posterior probability distributions. Subsamples are listed in parentheses; the first term denotes the population from which the activities were sourced, the latter denotes which population completed the likelihood ratings and other measures. ‘Relationship Quality’ refers to mean scores from the IPPA.

Supplementary Table 12. Associations between outcome variables and social value scores, controlling for unit-weighted likelihood ratings (Confirmatory sample, SONA-sourced activities).

Parent		Friend		Acquaintance	
		Relationship Quality			
Social Value Score - Parent	0.189 [0.11, 0.28]	Social Value Score - Friend	0.197 [0.14, 0.26]	Social Value Score – Acquaintance	0.174 [0.11, 0.24]
Unit Weighted Likelihood Rating - Parent	0.444 [0.36, 0.52]	Unit Weighted Likelihood Rating - Friend	0.226 [0.17, 0.28]	Unit Weighted Likelihood Rating – Acquaintance	0.262 [0.20, 0.32]
		Social Loss Aversion			
Social Value Score - Parent	1.794 [0.25, 3.13]	Social Value Score - Friend	1.307 [-0.01, 2.55]	Social Value Score – Acquaintance	1.787 [0.32 3.20]
Unit Weighted Likelihood Rating - Parent	2.779 [1.37, 4.24]	Unit Weighted Likelihood Rating - Friend	1.237 [0.89, 3.55]	Unit Weighted Likelihood Rating – Acquaintance	3.621 [2.18, 5.19]
		Dictator Game			
Social Value: Par - Fri	0.050 [0.01, 0.08]	Social Value: Par – Acq	0.012 [-0.02, 0.05]	Social Value: Fri – Acq	0.039 [0.01, 0.06]
Unit Weighted Likelihood: Par - Fri	0.077 [0.04, 0.12]	Unit Weighted Likelihood: Par - Acq	0.140 [0.10, 0.19]	Unit Weighted Likelihood: Fri - Acq	0.085 [0.05, 0.12]
		Forced Choice Time Spent			
Social Value: Par - Fri	0.475 [0.18, 0.77]	Social Value: Par – Acq	0.310 [-0.17, 0.79]	Social Value: Fri – Acq	0.382 [-0.28, 1.02]
Unit Weighted Likelihood: Par - Fri	0.822 [0.45, 1.18]	Unit Weighted Likelihood: Par - Acq	1.726 [1.11, 2.34]	Unit Weighted Likelihood: Fri - Acq	0.860 [0.07, 1.69]

Note. Bracketed numbers are 89% HDI posterior credible intervals. Each entry into the table is a coefficient from a regression model (logistic for the *forced choice time spent* item). Positive slopes for the first two outcomes (relationship quality, social loss aversion) indicate a direct relationship between the predictor and outcome. The predictors for the latter two outcomes (*dictator game* and *forced choice time spent*) are a difference score between social value scores

or unit weighted likelihood ratings between two known others. The label for such difference scores reflects the coding of the predictor; a positive value indicates a greater value for Person 1 relative to Person 2 and vice versa. As with other comparable analyses, outcomes were coded such that a positive slope for Person 1 means increases in Person 1's social value score were related to a preference for Person 1 over Person 2 for the given task; a negative slope for Person 2 means increases in Person 2's social value score were related to a preference for Person 2 over Person 1. These results reflect confirmatory phase data collected from prolific with SONA-sourced activities.

Parent		Friend		Acquaintance	
Relationship Quality					
Social Value Score - Parent	0.089 [0.00, 0.18]	Social Value Score - Friend	0.123 [0.07, 0.18]	Social Value Score – Acquaintance	0.039 [-0.04, 0.11]
Unit Weighted Likelihood Rating - Parent	0.430 [0.34, 0.52]	Unit Weighted Likelihood Rating - Friend	0.207 [0.15, 0.27]	Unit Weighted Likelihood Rating – Acquaintance	0.407 [0.33, 0.48]
Social Loss Aversion					
Social Value Score - Parent	1.419 [-0.06, 2.71]	Social Value Score - Friend	1.418 [0.16, 2.75]	Social Value Score – Acquaintance	1.078 [-0.39, 2.43]
Unit Weighted Likelihood Rating - Parent	2.589 [1.24, 3.94]	Unit Weighted Likelihood Rating - Friend	2.545 [1.12, 3.89]	Unit Weighted Likelihood Rating – Acquaintance	3.415 [1.99, 4.96]
Dictator Game					
Social Value: Par - Fri	0.026 [-0.01, 0.06]	Social Value: Par – Acq	0.024 [0.00, 0.05]	Social Value: Fri – Acq	0.035 [0.02, 0.06]
Unit Weighted Likelihood: Par - Fri	0.095 [0.06, 0.13]	Unit Weighted Likelihood: Par - Acq	0.086 [0.05, 0.12]	Unit Weighted Likelihood: Fri - Acq	0.062 [0.03, 0.09]
Forced Choice Time Spent					
Social Value: Par - Fri	0.106 [-0.16, 0.35]	Social Value: Par – Acq	0.094 [-0.21, 0.44]	Social Value: Fri – Acq	0.126 [-0.41, 0.66]
Unit Weighted Likelihood: Par - Fri	0.923 [0.56, 1.30]	Unit Weighted Likelihood: Par - Acq	1.236 [0.78, 1.72]	Unit Weighted Likelihood: Fri - Acq	1.484 [0.65, 2.27]

Supplementary Table 13. Associations between outcome variables and social value scores, controlling for unit-weighted likelihood ratings (Confirmatory sample, MTurk-sourced activities).

Note. Bracketed numbers are 89% HDI posterior credible intervals. Each entry into the table is a coefficient from a regression model (logistic for the *forced choice time spent* item). Positive slopes for the first two outcomes (relationship quality, social loss aversion) indicate a direct relationship between the predictor and outcome. The predictors for the latter two outcomes (*dictator game* and *forced choice time spent*) are a difference score between social value scores or unit weighted likelihood ratings between two known others. The label for such difference

scores reflects the coding of the predictor; a positive value indicates a greater value for Person 1 relative to Person 2 and vice versa. As with other comparable analyses, outcomes were coded such that a positive slope for Person 1 means increases in Person 1's social value score were related to a preference for Person 1 over Person 2 for the given task; a negative slope for Person 2 means increases in Person 2's social value score were related to a preference for Person 2 over Person 1. These results reflect confirmatory phase data collected from prolific with MTurk-sourced activities.

Supplementary Figure 1. Activity Weights

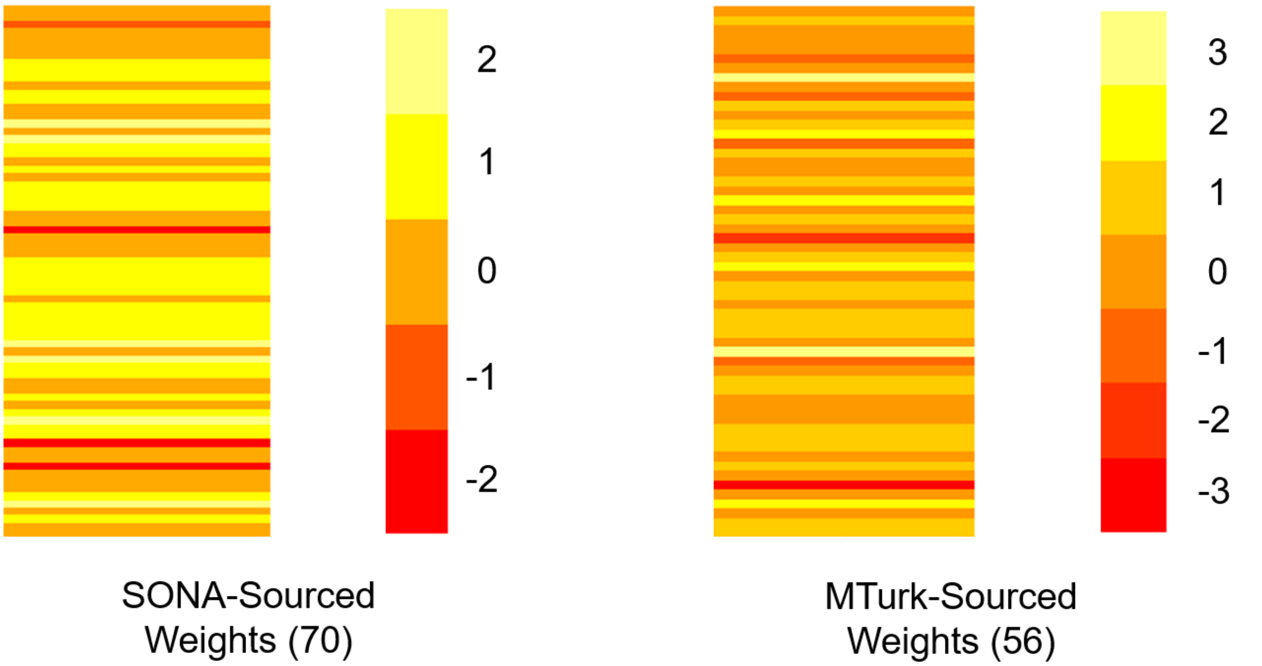

Note. SONA-Sourced Weights refers to weights of activities (*n* = 70 activities) that were sourced from the UCLA undergraduate psychology subject pool (SONA); Mturk-Sourced Weights refers to weights of activities (*n* = 56 activities) that were sourced from Amazon's Mechanical Turk. Note the range and variability in weights.

Supplementary Figure 2. Example trend of paired differences in social value scores between parents, friend, and acquaintances.

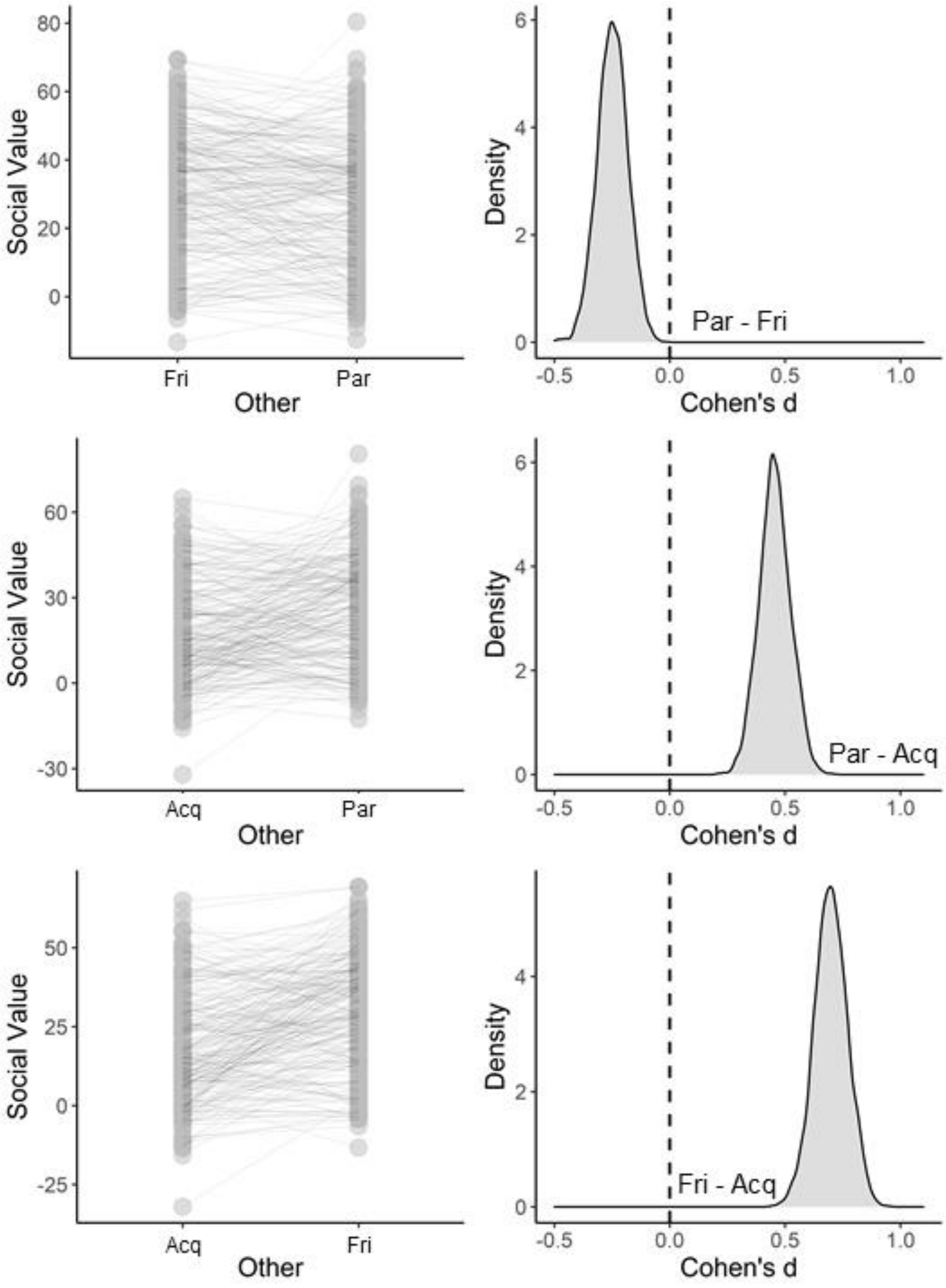

Note. The left column of plots shows paired differences between pairs of social partners. The right column depicts posterior distributions of paired differences (in Cohen's *d* metric). The hashed vertical line is centered over zero. Each column, respectively, shows paired differences between parent – friend, parent – acquaintance, and friend – acquaintance. Data for this visualization were drawn from confirmatory phase (prolific sample, SONA-sourced weights). 'Par' refers to parent, 'Fri' refers to friend, and 'Acq' refers to acquaintance. 'Density' refers to the mass of the posterior distribution. The sample size for this analysis is N = 233.

Supplementary Figure 3. Associations between social value scores and choice preferences when controlling for relationship quality and social loss aversion (Confirmatory sample, SONA-sourced activities).

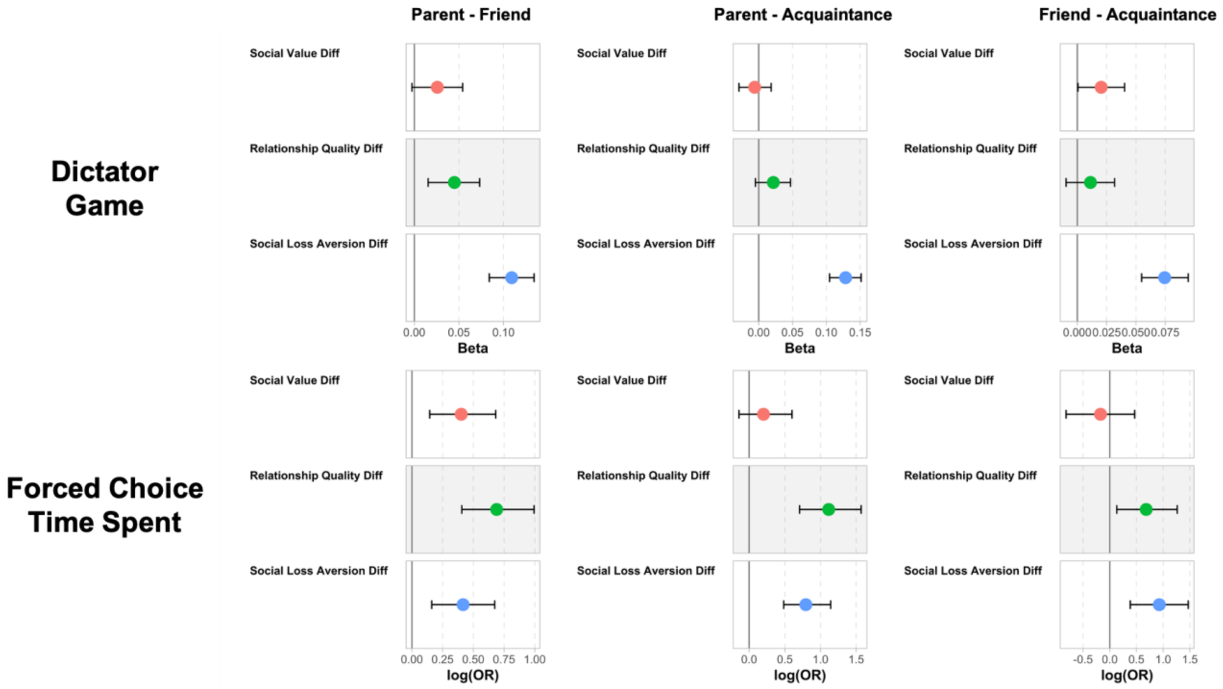

Note. ‘Forced Choice Spend Time’ refers to a one-shot, binary question asking participants to choose one of two social partners with whom they would rather spend a free afternoon. ‘Diff’ references to a difference score taken between the listed metric (social value scores, relationship quality, social loss aversion) for the two familiar others labeled at the top of each column (in the order listed, e.g., parent – friend means friend scores on a given metric were subtracted from parent scores). All predictors were standardized. Dots reflect coefficients from Bayesian regression and bars reflect 89% HDIs. Evidence was judged to be robust if the HDI did not include 0 or the HDI fell outside of the Region of Practical Equivalence (ROPE) and moderate if part of the HDI fell outside of ROPE (see “Inferential Criteria” section of the main text). ROPE was defined as the range between -0.1 to 0.1. Here, when controlling for relationship quality and social loss aversion, there was robust evidence that social value scores were associated with choice preferences regarding whether participants would rather spend time with their parent or friend and moderate evidence that social value scores were associated with choice preferences regarding whether participants would rather spend time with their parent or acquaintance and whether they would rather spend time with their friend or acquaintance. Comparable evidence was not found for choices in the dictator game. The sample size for this analysis is $N = 315$.

Supplementary Figure 4. Associations between social value scores and choice preferences when controlling for relationship quality and social loss aversion (Confirmatory sample, MTurk-sourced activities).

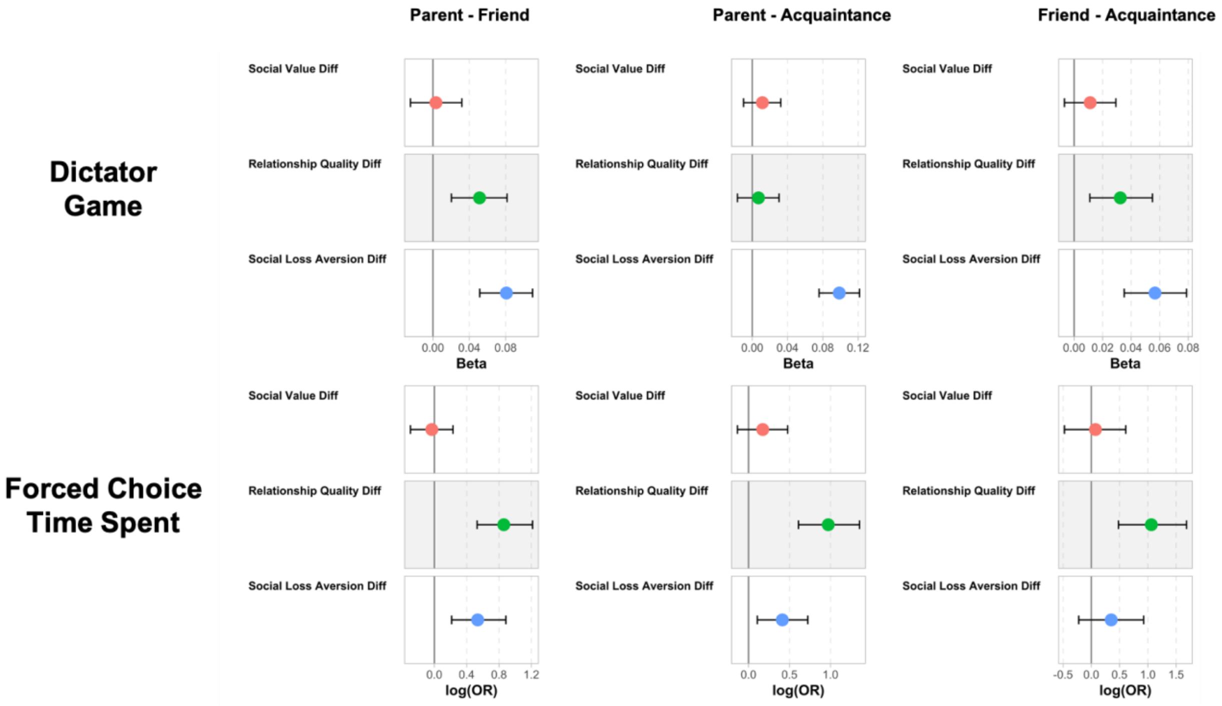

Note. 'Forced Choice Spend Time' refers to a one-shot, binary question asking participants to choose one of two social partners with whom they would rather spend a free afternoon. 'Diff' references to a difference score taken between the listed metric (social value scores, relationship quality, social loss aversion) for the two familiar others labeled at the top of each column (in the order listed, e.g., parent – friend means friend scores on a given metric were subtracted from parent scores). All predictors were standardized. Dots reflect coefficients from Bayesian regression and bars reflect 89% HDIs. Evidence was judged to be robust if the HDI did not include 0 or the HDI fell outside of the Region of Practical Equivalence (ROPE) and moderate if part of the HDI fell outside of ROPE (see "Inferential Criteria" section of the main text). ROPE was defined as the range between -0.1 to 0.1. Here, when controlling for relationship quality and social loss aversion, there was moderate evidence that social value scores were associated with choice preferences regarding whether participants would rather spend time with their parent or friend, whether they would rather spend time with their parent or acquaintance, and whether they would rather spend time with their friend or acquaintance. Comparable evidence was not found for choices in the dictator game. The sample size for this analysis is N = 320.
