# Supplementary material for: A behavioral signature for quantifying the social value of interpersonal relationships with specific others
Source: Commun Psychol. 2024 Sep 6;2:84. doi: 10.1038/s44271-024-00132-2 (PMC11379851; doi:10.1038/s44271-024-00132-2)
Supplement: Supplementary file 3 — Reporting Summary [file 44271_2024_132_MOESM3_ESM.pdf]

nature portfolio

Corresponding author(s): Joao F. Guassi Moreira

Last updated by author(s): Aug 23, 2024

Reporting Summary

Nature Portfolio wishes to improve the reproducibility of the work that we publish. This form provides structure for consistency and transparency in reporting. For further information on Nature Portfolio policies, see our Editorial Policies and the Editorial Policy Checklist.

Statistics

For all statistical analyses, confirm that the following items are present in the figure legend, table legend, main text, or Methods section.

n/a	Confirmed	
☐	☒	The exact sample size (*n*) for each experimental group/condition, given as a discrete number and unit of measurement
☐	☒	A statement on whether measurements were taken from distinct samples or whether the same sample was measured repeatedly
☐	☒	The statistical test(s) used AND whether they are one- or two-sided<br>*Only common tests should be described solely by name; describe more complex techniques in the Methods section.*
☐	☒	A description of all covariates tested
☐	☒	A description of any assumptions or corrections, such as tests of normality and adjustment for multiple comparisons
☐	☒	A full description of the statistical parameters including central tendency (e.g. means) or other basic estimates (e.g. regression coefficient) AND variation (e.g. standard deviation) or associated estimates of uncertainty (e.g. confidence intervals)
☒	☐	For null hypothesis testing, the test statistic (e.g. F, t, r) with confidence intervals, effect sizes, degrees of freedom and P value noted<br>*Give P values as exact values whenever suitable.*
☐	☒	For Bayesian analysis, information on the choice of priors and Markov chain Monte Carlo settings
☐	☒	For hierarchical and complex designs, identification of the appropriate level for tests and full reporting of outcomes
☐	☒	Estimates of effect sizes (e.g. Cohen's *d*, Pearson's *r*), indicating how they were calculated

Our web collection on statistics for biologists contains articles on many of the points above.

Software and code

Policy information about availability of computer code

Data collection	Qualtrics and PsychoPy were used to collect survey and task data, respectively. Surveys and task code is available at https://osf.io/rjqpc/
Data analysis	R was used for data analysis. All code is available at https://osf.io/rjqpc/

For manuscripts utilizing custom algorithms or software that are central to the research but not yet described in published literature, software must be made available to editors and reviewers. We strongly encourage code deposition in a community repository (e.g. GitHub). See the Nature Portfolio guidelines for submitting code & software for further information.

Data

Policy information about availability of data

All manuscripts must include a data availability statement. This statement should provide the following information, where applicable:

- Accession codes, unique identifiers, or web links for publicly available datasets
- A description of any restrictions on data availability
- For clinical datasets or third party data, please ensure that the statement adheres to our policy

All data are available for public download at: https://osf.io/rjqpc/

March 2021

Human research participants

Policy information about studies involving human research participants and Sex and Gender in Research.

Reporting on sex and gender	Biological sex and gender are reported in the manuscript.
Population characteristics	Age, ethnicity, and race are all reported in the manuscript
Recruitment	Participants were recruited through the UCLA undergraduate psychology subject pool, Amazon's Mechanical Turk, or Prolific
Ethics oversight	The Institutional Review Board at UCLA approved all study procedures.

Note that full information on the approval of the study protocol must also be provided in the manuscript.

Field-specific reporting

Please select the one below that is the best fit for your research. If you are not sure, read the appropriate sections before making your selection.

☐ Life sciences ☒ Behavioural & social sciences ☐ Ecological, evolutionary & environmental sciences

For a reference copy of the document with all sections, see nature.com/documents/nr-reporting-summary-flat.pdf

Behavioural & social sciences study design

All studies must disclose on these points even when the disclosure is negative.

Study description	Quantitative, correlational study
Research sample	UCLA Undergraduates, Online participants (MTurk, Prolific)
Sampling strategy	Convenience
Data collection	Data were collected via the online Qualtrics survey platform and/or Pavlovia.org
Timing	All data were collected between May and October of 2022.
Data exclusions	Data exclusion is detailed at length in the manuscript, but the gist is that certain data from MTurk were excluded if they appeared to be from a fraudulent responder. We abandoned MTurk because of such such responses and switched to Prolific mid-study.
Non-participation	None dropped out
Randomization	Participants were not sorted in random group. Certain questionnaire items and order of questionnaires or trials on computerized tasks were randomized us Qualtrics or PsychoPy

Reporting for specific materials, systems and methods

We require information from authors about some types of materials, experimental systems and methods used in many studies. Here, indicate whether each material, system or method listed is relevant to your study. If you are not sure if a list item applies to your research, read the appropriate section before selecting a response.

Materials & experimental systems

n/a	Involved in the study	
☒	☐	Antibodies
☒	☐	Eukaryotic cell lines
☒	☐	Palaeontology and archaeology
☒	☐	Animals and other organisms
☒	☐	Clinical data
☒	☐	Dual use research of concern

Methods

n/a	Involved in the study	
☒	☐	ChIP-seq
☒	☐	Flow cytometry
☒	☐	MRI-based neuroimaging
